# Scrolling into substance use: Social media use frequency and substance use among U.S. high school students

**DOI:** 10.1016/j.dadr.2026.100453

**Published:** 2026-06-05

**Authors:** Prabuddha Prakash, Francisco Beltran-Silva, Arjun Teotia

**Affiliations:** a Department of Economics, Rutgers University, NJ, USA; b Department of Economics, California State University Northridge, CA, USA; c Center for Health Policy and Health Services Research, Henry Ford Health System, MI, USA; d Department of Epidemiology and Biostatistics, Michigan State University College of Human Medicine, MI,USA

**Keywords:** Social Media, Substance Use, Adolescents, Platform Regulation, Influencer Marketing, Dose-Response

## Abstract

Adolescent substance use imposes lasting costs on education, mental health, and lifetime earnings, with early initiation strongly predicting adult dependence. Despite legal prohibition, approximately 21% of U.S. high school students report current alcohol use, 18% report current nicotine vaping, and 16% report current cannabis use. Social media plausibly contributes to these patterns, yet prior studies rely on broad screen time measures that conflate passive consumption with compulsive checking. We analyzed 2023 Youth Risk Behavior Survey (YRBS) data, the first standalone measure of social media frequency in the YRBS series, from 10,027 U.S. high school students. We estimated survey weighted Linear Probability Models with state fixed effects, adjusting for demographics, mental health, electronic bullying, sleep, and physical activity. Students checking social media more than once per hour show a 19 %age point (pp) higher probability of current alcohol use, a 16 pp higher probability of current nicotine vaping, and a 13 pp higher probability of current cannabis use relative to nonusers, following a dose response gradient. Applied to 17.1 million U.S. high school students, this associates with approximately 1 million additional students drinking alcohol, 870,000 additionally nicotine vaping, and 681,000 additionally using cannabis among the 5.4 million students checking social media more than once per hour. Associations concentrate in alcohol, cannabis, and nicotine vaping, which are prominently normalized online. These findings inform legislative debates over engagement maximizing design features targeting adolescents and support extending content restrictions on alcohol and cannabis influencer marketing.

**Background:**

Adolescent substance use imposes lasting costs on educational attainment, mental health, and lifetime earnings, with initiation during adolescence strongly predicting adult dependence. Despite legal prohibition, approximately 21% of U.S. high school students report current alcohol use, 18% report current nicotine vaping, and 16% report current cannabis use. Social media plausibly contributes to these patterns, yet most prior studies rely on composite screen time measures that conflate passive consumption with compulsive social media checking, limiting inference about frequency-specific risk.

**Methods:**

We analyzed 2023 Youth Risk Behavior Survey (YRBS) data, the first standalone measure of social media frequency in the YRBS series, comprising 10,027 U.S. high school students. We estimated survey-weighted Linear Probability Models with state fixed effects, adjusting for sociodemographic characteristics, mental health status, electronic bullying, sleep, and physical activity.

**Results:**

Students checking social media more than once per hour show 19 %age point (pp) higher probability of current alcohol use, a 16 pp higher probability of current nicotine vaping, and a 13 pp higher probability of current cannabis use relative to students reporting no use of social media, each following a dose-response gradient. Applied to 17.1 million U.S. high school students, these estimates associate with approximately 1 million additional students currently drinking alcohol, 870,000 additionally nicotine vaping, and 681,000 additionally using cannabis among the 5.4 million students checking social media more than once per hour.

**Conclusions:**

Associations between social media use frequency and substance use concentrate in alcohol, cannabis, and nicotine vaping, the three substances most prominently normalized in platform content, while lower for cigarettes which are subject to platform content bans and strong social stigma. These findings inform ongoing legislative debates over engagement-maximizing platform design features targeting adolescents and support extending content restrictions on alcohol and cannabis influencer marketing equivalent to those already applied to tobacco.

## Highlights

• Uses nationally representative social media frequency among U.S. high schoolers.
• Hourly or more frequent use associates with more alcohol, vaping, and cannabis use.
• Associations are strongest for substances marketed or normalized online.
• Findings support platform design regulation and influencer ad restrictions.

## 1 Introduction

Adolescent substance use imposes lasting costs on educational attainment, mental health, and lifetime earnings, with initiation during adolescence strongly predicting adult dependence and psychiatric comorbidity (CDC, 2025, Miech et al., 2024). In our sample, 21% of U.S. high school students report current alcohol use, 18% report current nicotine vaping, and 16% report current cannabis use, despite legal prohibitions for this age group. The rise of social media, with its peer-generated content and algorithmically amplified exposure, has introduced an unprecedented channel through which substance use behaviors are observed and normalized in adolescent daily life. Approximately one third of U.S. high school students constantly use social media (Faverio and Sidoti, 2024), making compulsive platform engagement a near-universal feature of adolescent experience (Rideout, 2021). This study examines whether social media use frequency associates with substance use among U.S. high school students.

Social learning theory predicts that individuals adopt behaviors they observe others performing, particularly when those behaviors appear prevalent and positively reinforced by peers (Boone et al., 1977). Social media amplifies this mechanism by delivering algorithmically personalized, peer-generated content at a volume and frequency no prior medium could match (Primack et al., 2015). Crucially, the frequency with which an adolescent returns to a platform determines the cumulative normative exposure. A teenager checking once a day accumulates fundamentally different content than one checking more than once per hour. The 2023 Surgeon General’s Advisory on Social Media and Youth Mental Health identified compulsive, high-frequency social media use as a distinct risk profile for adolescent health warranting targeted attention (Office of the Surgeon General (OSG), 2023).

A growing body of research links social media use to adolescent substance use across multiple substances. Experimental evidence shows that brief exposure to alcohol-related influencer content increases use intentions among adolescents (Potvin Kent et al., 2025). Longitudinal evidence from a nationally representative UK cohort finds that social media use at age 14 increases the risk of alcohol use and binge drinking at age 17, with associations following a frequency gradient (Purba et al., 2023). Among U.S. adolescents, longitudinal evidence shows that frequent exposure to nicotine e-cigarette and cannabis posts from influencers on TikTok, Instagram, and YouTube associates with initiation of both substances at follow-up (Vassey, Chen-Sankey, et al., 2025). Evidence is less consistent for cigarettes, where platform content policies and social stigma differ markedly from alcohol, cannabis, and nicotine vaping (Rutherford et al., 2023). Most population level studies rely on coarse screen time measures aggregating television, video games, and internet use, conflating passive consumption with the peer-facing social engagement characteristic of social media (Shannon et al., 2022). No prior study using nationally representative U.S. data has examined the full gradient from low to compulsive social media use frequency across multiple substance outcomes simultaneously, making it impossible to assess whether associations are broad or substance-specific (Galea et al., 2024). Additionally, studies rarely adjust for the full set of psychosocial risk factors, including electronic bullying, physical activity, and sleep disruption, that are independently associated with both heavy social media use and substance use (Orben and Przybylski, 2019).

Although a rich body of literature establishes a link between social media and adolescent tobacco/cannabis use (Donaldson et al., 2025, Lee et al., 2023a, Lee et al., 2023b, Richardson et al., 2024, Zheng et al., 2021, Zheng et al., 2024), assessing this relationship across a broader spectrum of substances remains methodologically challenging. Existing research frequently relies on datasets optimized for specific outcomes or populations: the Population Assessment of Tobacco and Health (PATH) survey is primarily designed for adult tobacco regulation, limiting generalizability to the broader adolescent population (Hyland et al., 2017); the National Youth Tobacco Survey (NYTS) focuses on tobacco, excluding prevalent substances like current alcohol or cannabis use, and lacks key psychosocial covariates; Adolescent Behaviors and Experiences Survey (ABES) used previously by (Shockey and Silver, 2025) bundle social media into composite screen-time measures; the Adolescent Brain Cognitive Development (ABCD) data provides detailed longitudinal screen use measures from ages 9–13 and captures substance use primarily as lifetime initiation events at this developmental stage (Sullivan et al., 2022). The present study complements this work by examining current 30-day use in the high school years, when substance use rises sharply and policy debates over platform regulation concentrate. This study addresses these limitations by utilizing the 2023 Youth Risk Behavior Survey (YRBS). The 2023 YRBS introduces a standalone, dedicated measure of social media frequency alongside outcomes for four major substances (alcohol, cannabis, nicotine vaping, and cigarettes) and a comprehensive set of psychosocial confounders. By standardizing these measures within a single nationally representative high school sample, this study aims to estimate substance-specific dose-response. Recent work by (Nagata et al., 2025) links screen time to cannabis initiation. The present study offers a complementary perspective by evaluating current (past 30-day) use across four distinct substances and correlates with past 30-day substance use behaviors. Because substance use prevalence varies significantly across demographic groups (Miech et al., 2024), and social media algorithms frequently deliver demographically targeted, highly personalized content streams (Chen-Sankey et al., 2019), it is crucial to understand if digital normalization operates uniformly. Therefore, we examine heterogeneous associations by sex and race/ethnicity to ensure pooled estimates do not obscure concentrated vulnerabilities in specific subpopulations. Motivated by (Wang et al., 2025) we explore whether physical activity moderates these associations, serving as an exploratory test of whether structured, offline routines buffer the negative impacts of frequent social media use.

## 2 Methods

### 2.1 Study design and data source

This study uses data from the restricted-use 2023 National YRBS, the first standalone measure of social media frequency in the YRBS series. We obtained access to the dataset including state identifiers via a request to the Centers for Disease Control and Prevention (CDC). The restricted version of YRBS allows identification of states based on school location, enabling control for state-level heterogeneity. The YRBS uses a three-stage cluster sampling design to produce nationally representative estimates for students in grades 9 through 12 who attend public and private schools. Full details of survey design, sampling procedures, and data collection are provided in the CDC's 2023 YRBS Data User's Guide (CDC, 2025). Of 15, 203 students with non-missing data on the social media exposure variable, 10,027 had complete data on all four substance use outcomes and the full covariate set. We applied listwise deletion to retain a uniform analytic sample across all four outcome models*.* This study used de-identified secondary data from the YRBS and was exempt from Institutional Review Board review.

### 2.2 Primary exposure: social media use frequency

The 2023 YRBS introduced a new granular measure for social media engagement. Prior to 2023, the YRBS measured screen time as a composite aggregate of television, video games, and internet use, designed to assess sedentary behavior rather than digital engagement. This shift allows us to capture compulsive social media use, which differs from prolonged but passive consumption. Social media use is measured using the question, "How often do you use social media?" Response options include: "I do not use social media (1)," "A few times a month (2)," "About once a week (3)," "A few times a week (4)," "About once a day (5)," "Several times a day (6),” “About once an hour (7),” and “More than once an hour (8)." The original 8-point social media frequency question is collapsed into a 5-level variable by isolating the absolute lowest and highest responses and pairing the adjacent intermediate categories (values 2 & 3, 4 & 5, and 6 & 7) to prevent the data from being spread too thin. We categorize responses into five levels: (a) no use; (b) a few times per month or week; (c) a few times per week to once per day; (d) several times per day to about once per hour; and (e) more than once per hour.

### 2.3 Outcomes

We examined four binary substance use outcomes, all drawn from the 2023 YRBS questionnaire. Current cigarette use is derived from the question: "During the past 30 days, on how many days did you smoke cigarettes?" Students reporting one or more days are coded as individuals currently using cigarettes . Current nicotine vapor product use is derived from: "During the past 30 days, on how many days did you use an electronic vapor product?" Current alcohol use is derived from: "During the past 30 days, on how many days did you have at least one drink of alcohol?" Current cannabis use is derived from: "During the past 30 days, how many times did you use marijuana?" All four outcomes are coded as binary indicators.

### 2.4 Covariates

The covariate set includes sex, age, race and ethnicity (Non-Hispanic White, Non-Hispanic Black, Hispanic, and Other including Indigenous, Asian, or Pacific Islander), mental health status (1 if past−30 day poor, “most of the time” or “always”), physical activity (1 if 60 + minutes on 5 + days a week), electronic bullying victimization (1 if any in past 12 months), and sleep (1 if 8 + hours on an average school night). These covariates are selected to mitigate omitted variable bias by controlling for underlying psychosocial distress and structured routines that can influence both social media engagement and substance use. State fixed effects are included in each model to control for unobserved state-level heterogeneity in substance policies, cultural norms, and economic conditions, restricting comparisons to students within the same state.

### 2.5 Statistical analysis

We first summarized the distribution of social media use and outcome variables in the sample, applying survey weights to account for the complex sampling design. Linear Probability Model (LPM) is then used to estimate the association between social media use frequency and each substance use outcome, with no use as the reference category. Survey design-based standard errors accounted for the complex cluster sampling design using the centered single-PSU adjustment. All analyses were conducted in Stata version 19. To examine the sensitivity of the results to the use of LPM, we estimated survey-weighted logistic models and interactions with physical activity. Because four separate substance use outcomes are evaluated using the same independent variables, a Bonferroni correction is applied to control for the Family-Wise Error Rate (FWER). Thus, the threshold for statistical significance across all four regression models was adjusted to 0.05/4.

## 3 Results

### 3.1 Sample characteristics

Table 1 presents descriptive statistics for the analytic sample. The sample includes 10,027 students after excluding missing observations on covariates and mental health status. Current nicotine vaping is prevalent in 18%, current alcohol use in 21%, current cannabis in 16%, while current cigarette in 4% of students. A total of 46% of students used social media several times per day to about once per hour, and 32% used it more than once per hour. 7% reported no social media use. Females comprised 50% of the sample and 31% of students reported poor mental health.

**Table 1:** Summary Statistics from the 2023 YRBS (N = 10,027).

**Variable**	**Mean**	**Std. Dev.**
**Outcomes**		
Current cigarette use	0.037	0.19
Current nicotine vape use	0.18	0.38
Current alcohol use	0.21	0.41
Current cannabis use	0.16	0.37
Any Illicit Drug	0.15	0.36
**Social Media Use Frequency**		
No use	0.071	0.26
Few per month/week	0.042	0.20
Few per week to daily	0.11	0.31
Several per day to hourly	0.46	0.50
More than once per hour	0.32	0.47
**Covariates**		
Mental health (poor)	0.31	0.46
Physical activity	0.70	0.46
Sex (female)	0.50	0.50
Race: Hispanic	0.20	0.40
Race: Non-Hispanic Black	0.091	0.29
Race: Non-Hispanic White	0.49	0.50
Race: Other/Multiracial	0.22	0.41
Bullied electronically	0.16	0.37
Sleep 8 or more hours	0.057	0.23

Notes: Survey-weighted means and standard deviations. Sample comprises U.S. high school students with complete data on all analytic variables.

### 3.2 Social media use frequency and substance use

Table 2 reports survey-weighted LPM estimates for four substance use outcomes. Column 1 shows the results for current cigarette use. Students using social media several times per day to hourly are correlated with a 2 %age point (pp) higher probability (p < 0.012) of current cigarette use relative to those reporting no use. Students using social media more than once per hour link to a 5 pp higher probability (p < 0.0025). Column 2 shows the results for current nicotine vaping. Associations are not significant for lower than daily social media use. Students using social media several times per day to hourly link to an 8 pp higher probability (p < 0.0025) of current nicotine vaping relative to those reporting no use. Students using social media more than once per hour are correlated with a 16 pp higher probability (p < 0.0025). Column 3 shows the results for current alcohol use. Associations were insignificant for lower than daily social media use. Students using social media several times per day to hourly link to a 12 pp higher probability (p < 0.0025). Students using social media more than once per hour link to a 19 pp higher probability (p < 0.0025). Column 4 shows the results for current cannabis use. As seen for cigarettes and nicotine vaping, associations are not significant for lower than daily social media use. Students using social media several times per day to hourly associate with a 9 pp higher probability (p < 0.0025) of current cannabis use relative to those reporting no use. Students using social media more than once per hour associate with a 13 pp higher probability (p < 0.0025).

**Table 2:** Social Media Use Frequency and Substance Use.

	**(1)**	**(2)**	**(3)**	**(4)**
	**Cigarettes**	**Nicotine Vape**	**Alcohol**	**Cannabis**
Few per month/week	0.0038 [−0.0092, 0.099]	0.0024 [−0.037, 0.041]	0.055 [−0.0018, 0.11]	−0.0021 [−0.043, 0.039]
	(0.0049)	(0.020)	(0.029)	(0.021)
Few per week/day	0.016 [−0.00020, 0.031]	0.021 [−0.011, 0.052]	0.048 [0.0082, 0.087]	0.025 [−0.0094, 0.058]
	(0.0080)	(0.016)	(0.020)	(0.017)
Several/day to hourly	0.018** [0.0066, 0.030]	0.084*** [0.057, 0.11]	0.12*** [0.092, 0.16]	0.090*** [0.061, 0.12]
	(0.0059)	(0.014)	(0.017)	(0.015)
More than hourly	0.048*** [0.030, 0.066]	0.16*** [0.13, 0.19]	0.19*** [0.15, 0.22]	0.13*** [0.093, 0.16]
	(0.0092)	(0.017)	(0.019)	(0.017)
N	10,027	10,027	10,027	10,027

Notes: Survey design-based standard errors in parentheses. 95% confidence intervals are included in the square brackets alongside the estimates. All models include state fixed effects, and the full covariate set. Reference category: no social media use. Bonferroni-adjusted significance thresholds: * p < 0.025, ** p < 0.012, *** p < 0.0025.

### 3.3 Heterogeneity by sex and race and ethnicity

**Fig. 1:**
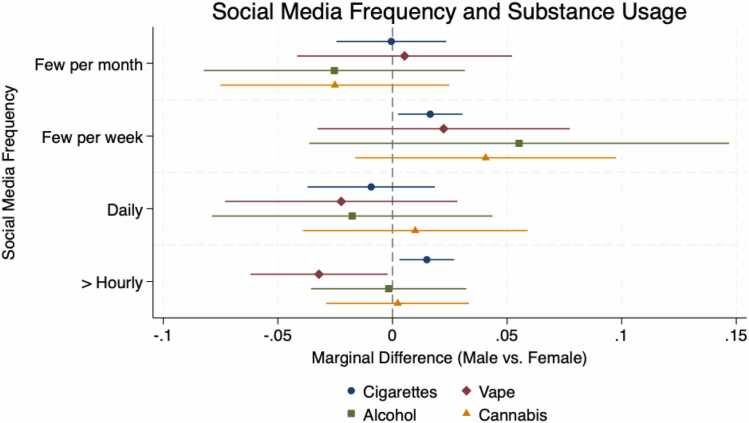
**Heterogeneity by Sex: Association between Social Media Use and Substance Use,** Legend: Coefficients represent the interaction term (Social Media Frequency × Male). All models have 10,027 observations. Markers: Circle =  Cigarette; Diamond =  Nicotine Vape; Square =  Alcohol; Triangle =  Cannabis. The dashed line at 0 indicates no effect.

**Fig. 2:**
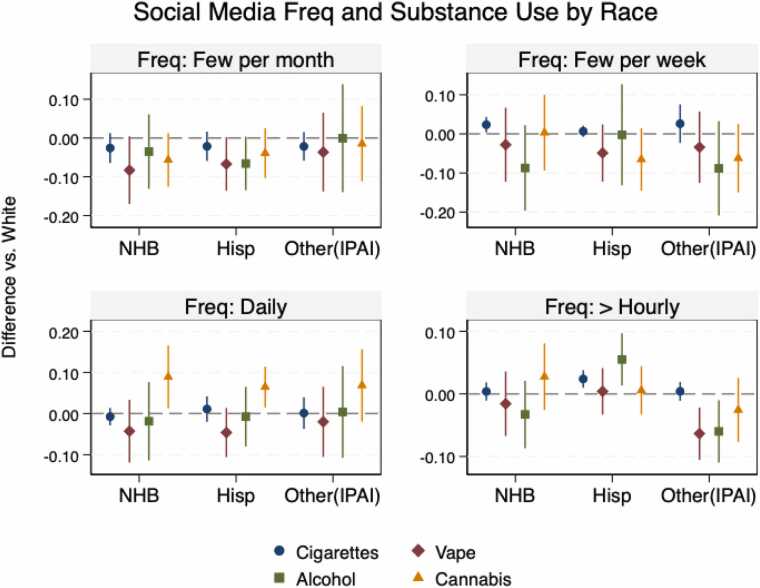
**Heterogeneity by Race: Association between Social Media Use and Substance Use,** Legend: Coefficients represent the interaction term (Social Media Frequency × Race). All models have 10,027 observations. Markers: Circle =  Cigarette; Diamond =  Nicotine Vape; Square =  Alcohol; Triangle =  Cannabis. The dashed line at 0 indicates no effect.

Fig. 1 examines heterogeneity by sex. Point estimates hover around zero, indicating associations are not statistically different for males and females. Fig. 2 presents heterogeneity by race and ethnicity, with Non-Hispanic White students as the reference. Across most substances and social media use frequencies (few per month, few per week, and daily), the point estimates largely hover around zero with confidence intervals crossing the zero line, indicating that these associations are generally not statistically different from those of Non-Hispanic White students. However, at the highest frequency of social media use (hourly or more), some differences emerge. Hispanic students exhibit a stronger association with alcohol use compared to White students, while students in the other race categories show weaker associations of nicotine vaping and alcohol use.

### 3.4 Sensitivity checks

**Figure A1:**
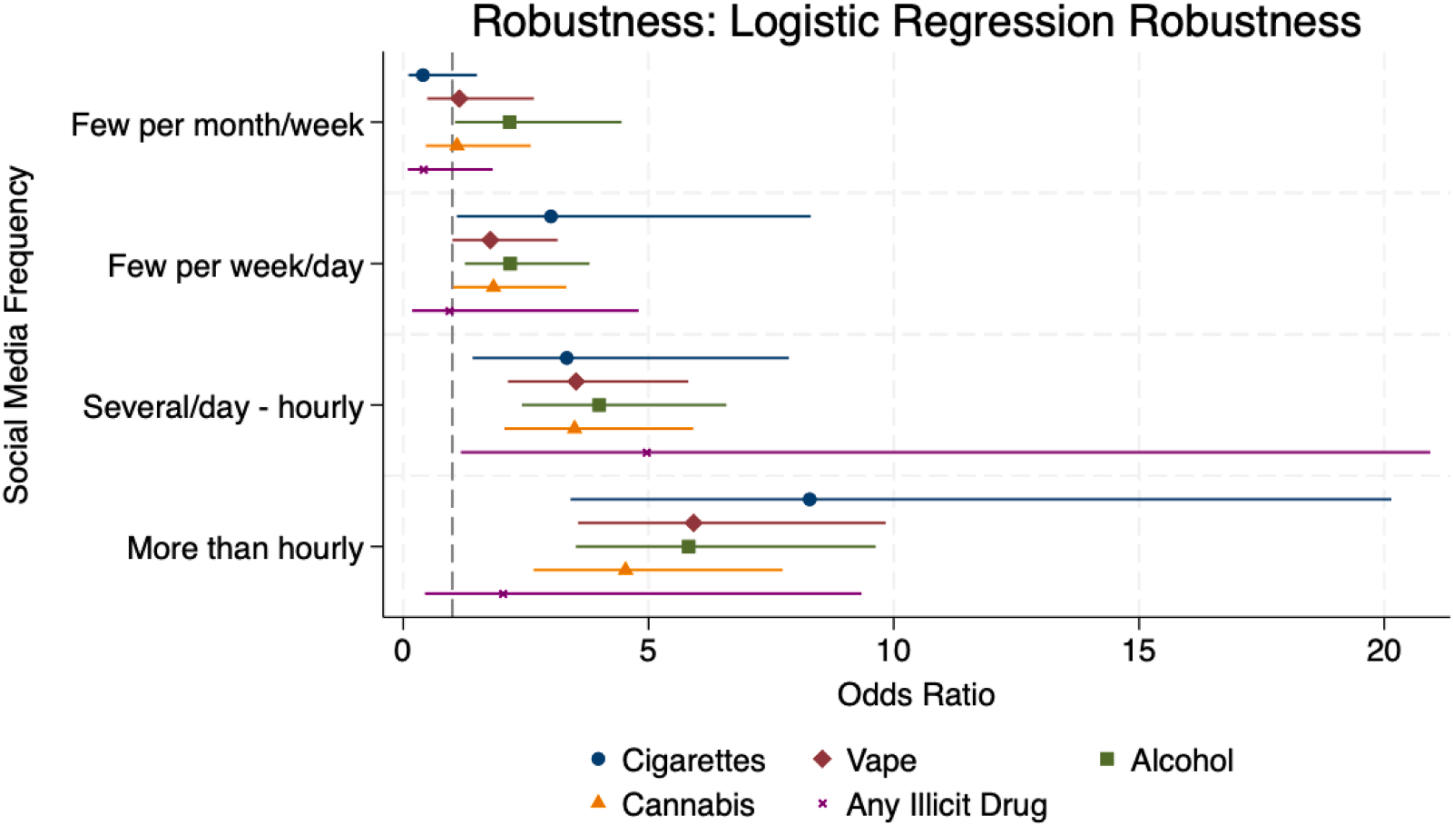
**Robustness Check by calculating Odds Ratios from the Logistic Regression.**, Legend: Estimates represent Odds Ratios (OR) rather than linear coefficients. All the models have 10,027 observations. Markers: Circle =  Cigarette; Diamond =  Vape; Square =  Alcohol; Triangle =  Cannabis; Cross =  Any Illicit Drug. The dashed line at 1 indicates no effect

**Figure A2:**
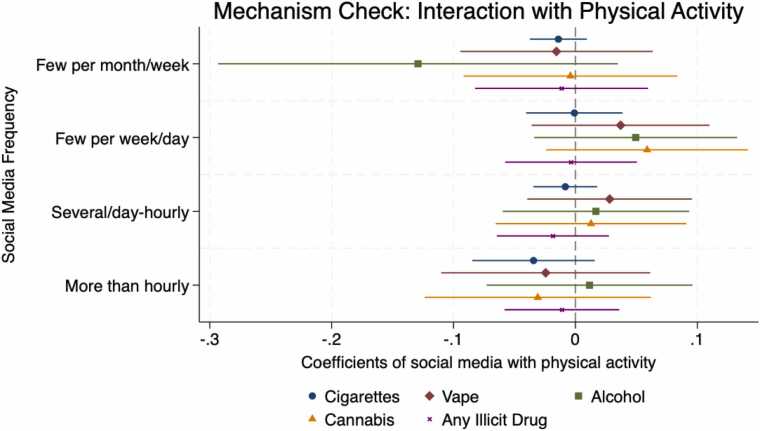
**Mechanism Check: Interaction with Physical Activity**, Legend: Estimates represent linear probability models coefficients. All the models have 10,027 observations. Markers: Circle =  Cigarette; Diamond =  Vape; Square =  Alcohol; Triangle =  Cannabis; Cross =  Any Illicit Drug. The dashed line at 0 indicates no effect

We replicated the analysis using survey-weighted logistic regression to verify that findings were robust to model specification. Results were consistent with the main estimates in direction and significance across all four outcomes (see Appendix Figure A1). We also examined whether physical activity moderates the association between social media use and substance use. Interaction terms were not statistically significant across any outcome (see Appendix Figure A2).

YRBS also reports on additional illicit substances, specifically cocaine, heroin, ecstasy, inhalants, methamphetamines, and hallucinogens. We analyzed a composite indicator for these substances and the Appendix Table A1 shows this check. Coefficients were small and statistically insignificant across all frequency categories. This model is analyzed separately from our main models because the YRBS captures these additional substances using a lifetime recall window, which differs methodologically from the 30-day 'current use' window used for our four primary outcomes. While LPM serves as our primary specification due to the interpretation of their coefficients, we also estimated survey-weighted logistic models to robustness of our results to the linear functional form. Lastly, drawing on (Wang et al., 2025), we introduce interaction terms with physical activity as a test to assess whether offline activities moderate the negative impacts of social media usage.

## 4 Discussion

### 4.1 Main findings

This study examines the association between social media use frequency and four substance use outcomes among U.S. high school students using the 2023 YRBS. Students checking social media more than once per hour are correlated with a 19 pp higher probability of current alcohol use, a 16 pp higher probability of current nicotine vaping, and a 13 pp higher probability of current cannabis use relative to those reporting no use. Each association follows a dose-response gradient, strengthening monotonically across frequency tiers. Applied to 17.1 million U.S. high school students, these estimates translate to approximately 1 million additional students drinking alcohol, 870,000 nicotine vaping, and 681,000 using cannabis among the 5.4 million students checking social media more than once per hour. Cigarette use shows a smaller but significant association, with a 5 pp higher probability at more than hourly social media use.

### 4.2 Comparison to prior literature

These findings extend prior work linking social media use to adolescent substance use (Rutherford et al., 2023, Vannucci et al., 2020, Vassey et al., 2025) in three ways. First, we apply a granular, nationally representative measure of social media use frequency. Second, the dose-response gradient we observe is consistent with cumulative social learning theory (Boone et al., 1977). Each return to the platform represents an additional exposure to substance-positive content. A student checking once a day accumulates fundamentally different normative content than one checking more than once per hour. Third, we examine four substance outcomes simultaneously in a single nationally representative sample. This design reveals that associations concentrate in alcohol, cannabis, and nicotine vaping while smaller for cigarettes, a substance-specific pattern that carries direct implications for understanding which platform content environments drive adolescent use.

### 4.3 The normalization mechanism

The substance-specific pattern of associations is consistent with social learning theory, which predicts that individuals adopt behaviors they observe peers and aspirational figures performing, particularly when those behaviors appear prevalent and positively reinforced (Boone et al., 1977). Social media platforms do not treat all substances equally. Alcohol, cannabis, and nicotine vaping are embedded in aspirational lifestyle content across Instagram, TikTok, and YouTube, with influencer posts portraying use as social, desirable, and routine (Jung et al., 2024, Potvin Kent et al., 2025, Primack et al., 2015). A systematic review of substance-related posts across major platforms found that 76% were depicted positively, with that positivity concentrated in alcohol, cannabis, and nicotine vaping content (Rutherford et al., 2023). Longitudinal evidence from over 7600 California high school students shows that adolescents exposed to cannabis and nicotine vaping influencer posts on these platforms were significantly more likely to subsequently initiate use (Vassey, Chen-Sankey, et al., 2025). Our dose-response gradient for these three substances aligns with what cumulative exposure predicts. Cigarette findings are equally consistent with this framework. Decades of tobacco denormalization have made cigarette smoking socially stigmatized among U.S. adolescents, and platforms actively suppress tobacco content (Bell et al., 2010, Laestadius et al., 2019). The contrast between cigarettes and nicotine vaping is particularly instructive. Nicotine vaping brands have invested heavily in influencer marketing on TikTok and Instagram, deliberately embedding use in humor, music, and peer social contexts (Laestadius et al., 2019). Our analysis reflects the divergence that nicotine vaping associates at 16 pp at more than hourly use while cigarettes associate at 5 pp. The absence of significant racial/ethnic heterogeneity is consistent with the platform-wide reach of normalizing content. Algorithmic delivery of substance-positive content operates across demographic groups, and our estimates suggest the normalization mechanism does not concentrate in any racial or ethnic subgroup.

### 4.4 Policy implications

These findings speak directly to ongoing legislative debates over youth digital safety. The (*Kids Online Safety Act | U.S. Senator Richard Blumenthal of Connecticut*, 2025), which passed the U.S. Senate 91–3 in July 2024 and was reintroduced in the 119th Congress in May 2025, would require platforms to disable design features that drive compulsive use among minors, explicitly naming infinite scroll, autoplay, push notifications, and rewards for time spent as features resulting in compulsive checking behavior (*Kids Online Safety Act | U.S. Senator Richard Blumenthal of Connecticut*, 2025). Australia enacted a nationwide restriction on social media access for children under 16 in November 2024 (Department of Infrastructure, 2025). Our results are consistent with the usage patterns these legislative efforts aim to address - associations with alcohol, nicotine vaping, and cannabis concentrate precisely at the hourly and more-than-hourly frequency tiers that engagement-maximizing platform architecture is engineered to produce. The 2023 Surgeon General's Advisory identified this compulsive, high-frequency use profile as a distinct clinical risk warranting policy attention (Office of the Surgeon General (OSG), 2023).

The cross-substance pattern in our analysis carries a targeted policy implication beyond engagement regulation. Platforms currently enforce content bans on tobacco promotion, which is the substance showing the smallest or null associations in our data. Alcohol and cannabis influencer marketing operates largely without equivalent restriction. Sponsored posts by alcohol brands and cannabis lifestyle influencers reach adolescent audiences through algorithmic amplification, often without clear disclosure. The Federal Trade Commission updated its Endorsement Guides in July 2023, flagging child-directed advertising as an area of heightened concern and requiring clear, conspicuous disclosure of material connections between influencers and brands (16 CFR Part 255 -- Guides Concerning Use of Endorsements and Testimonials in Advertising, n.d.). Enforcement targeting undisclosed alcohol and cannabis promotion reaching minor audiences remains limited. Extending platform content restrictions to alcohol and cannabis influencer marketing, consistent with the tobacco content restrictions already in place, would address a mechanism consistent with the patterns our data document.

Regulating the structural features that drive compulsive checking, rather than targeting individual adolescent behavior, represents a more tractable and equitable intervention. Adolescents do not choose the algorithmic architecture that shapes their feeds. Policy directed at platforms addresses the source of exposure rather than placing the responsibility of harm reduction on the adolescents most affected.

Translating these findings into effective practice requires a multi-level prevention framework, consistent with guidance from the American Academy of Pediatrics’ Center of Excellence on Social Media and Youth Mental Health (American Academy of Pediatrics, 2024). At the family level, utilizing tools such as the Family Media Plan and the 5Cs (Child, Content, Calm, Crowding-out, Communication) framework can help parents co-create age-appropriate digital boundaries and facilitate critical discussions regarding online substance exposure (Moreno, 2014, Stevens et al., 2020). Within educational settings, school-based media literacy curricula should explicitly address digital marketing tactics, influencer disclosure rules, and algorithmic amplification to build adolescent resilience against normalized substance content (Donaldson et al., 2022, Donaldson et al., 2025, Vogel et al., 2025). Finally, peer-led prevention programs could offer a critical complementary approach (Curtis et al., 2018, Lim et al., 2024, Trangenstein et al., 2019).

Public health campaigns operate within a fragmented digital media landscape. While targeted digital messaging and influencer partnerships have been associated with greater youth awareness of e-cigarette harms (MacMonegle et al., 2024), platform policies often do not restrict sponsored influencer content, implement age verification, or consistently enforce tobacco-related guidelines (Kong et al., 2024). Social media discourse describe strategies to work around tobacco control policies, including illicit online markets and international imports (Dobbs et al., 2026). Together, these dynamics shape the content environment surrounding adolescent social media use.

### 4.5 Limitations

This study has several limitations. First, the findings from the study should be seen as correlational rather than causal. Adolescents with greater propensity for substance use may select into higher social media use, and longitudinal data would help distinguish selection from exposure effects. The cross-sectional design also cannot rule out reverse causation or bidirectional relationships, in which substance use itself increases engagement with substance-related content that platforms then amplify through algorithmic recommendation. Second, similar to (Nagata et al., 2025), it is important to note that our measure captures the frequency of platform returns rather than active engagement, such as liking, sharing, or viewing substance-specific content and should be a priority of the future research. We observe that associations map onto documented content asymmetries across substances, but we cannot directly link individual adolescents to the specific content they encounter. Future research linking frequency measures to content exposure data would sharpen mechanistic inference. Third, the 2023 YRBS introduced this social media frequency measure for the first time, replacing a composite screen time item that combined television, video games, and internet use. This prevents direct comparison with prior survey waves and limits assessment of temporal trends. Several future waves using this identical frequency item will be necessary to establish whether these associations are changing over time. Fourth, while we adjust for a comprehensive set of sociodemographic, behavioral, and family risk factors, unmeasured confounders including peer norms, parental attitudes toward substance use, and prior substance use history may influence observed associations. Lastly, our findings apply to U.S. high school students in grades 9–12 (ages approximately 12–18). Whether similar dose-response patterns hold for younger adolescents and young adults is an important question for future research using complementary datasets such as ABCD and Monitoring the Future.

## 5 Conclusions

This study found that social media use frequency associates with alcohol use, nicotine vaping, and cannabis use among U.S. high school students, with strongest associations concentrated at the two highest frequency tiers. Associations were smaller and significant for cigarette use, a cross-substance pattern that maps directly onto documented asymmetries in how platforms portray each substance. Future research should examine whether these patterns persist across subsequent YRBS waves and whether direct measures of content exposure explain these differences. These findings support continued efforts targeting engagement-maximizing platform design and substance-related influencer marketing as potential targets for adolescent substance use prevention.

## CRediT authorship contribution statement

**Arjun Teotia:** Writing – review & editing, Supervision, Data curation, Conceptualization. **Francisco Beltran-Silva:** Writing – review & editing, Supervision. **Prabuddha Prakash:** Writing – original draft, Software, Formal analysis.

## Funding/support

None reported.

## Role of the funder/sponsor

None reported.

## Declaration of Competing Interest

I, and all the other authors declare that we have no relevant or material financial interests that relate to the research described in this paper.

## Figures and Tables

**Table A1:** Social Media Use Frequency and Illicit Drug Usage

	**(1)**
	**Any Illicit Drug**
Few per month/week	−0.0086 [−0.064, 0.047]
	(0.028)
Few per week/day	0.00044 [−0.040, 0.041]
	(0.021)
Several/day to hourly	0.015 [−0.021, 0.051]
	(0.018)
More than hourly	0.031 [−0.0073, 0.070]
	(0.020)
N	10,027

Notes: Survey design-based standard errors in parentheses. “Any Illicit Drug” is a binary variable which takes value 1 if the respondent answers a yes to having every used any of the illicit drug substances. 95% confidence intervals are included in the square brackets alongside the estimates. All models include state fixed effects, and the full covariate set. Reference category: no social media use. * p < 0.10, ** p < 0.050, *** p < 0.010.

## Data Availability

YRBS data were used for this analysis and are available publicly or by request to the CDC.
